# Laboratory-Based Investigation into Stress Corrosion Cracking of Cable Bolts

**DOI:** 10.3390/ma12132146

**Published:** 2019-07-03

**Authors:** Saisai Wu, Jinping Guo, Guangbin Shi, Junping Li, Caiwu Lu

**Affiliations:** 1 School of Resources Engineering, Shanxi Key Laboratory of Geotechnical and Underground Space Engineering, XAUAT, Xian 710055, China; 2 International Joint Research Laboratory of Henan Province for Underground Space Development and Disaster Prevention, Henan Polytechnic University, Jiaozuo 454003, China; 3 School of Minerals and Energy Resources Engineering, UNSW Sydney, Sydney, NSW 2052, Australia

**Keywords:** stress corrosion cracking, cable bolts, reinforcement system, hydrogen embrittlement

## Abstract

Cable-bolt failures due to stress corrosion cracking (SCC) could significantly compromise the sustainability and long-term stability of underground constructions. To fully understand the SCC of cable bolts, a two-step methodology was implemented: (i) long-term cable-bolt coupon tests using mineralogical materials collected from underground mines; and (ii) accelerated full-scale cable-bolt tests using an acidified solution. In the long-term tests, a novel three-point bending coupon was designed. The effects of mineralogical materials on SCC were evaluated under the simulated underground bolting conditions through the application of “corrosion cells”. For accelerated tests, SCC resistance of different type of cable bolts was examined using the new designed tensile-loading apparatus under the periodically increasing strain-rate loading mechanism. It was identified that mineralogical materials and applied stress intensity accelerated the corrosion process of the cable bolts. The number of wires and wire surface conditions in different types of cable bolt directly affected SCC susceptibility. The cable bolts with a greater number of wires provided higher resistance to SCC. The developed experimental methodologies can be applied to study SCC in other reinforcement materials and the results can be used to design optimal support systems in different environmental and geotechnical conditions.

## 1. Introduction

In mining and civil engineering, the construction of the underground excavations such as roadways and tunnels disturbs the original stabilization of the rock mass. The stresses around the excavations redistribute both vertically and horizontally which can cause the fracture and collapse of the strata, potentially leading to the injury of the human and loss of equipment [1,2]. To reinforce the rock mass around the excavations and prevent the strata from collapse, cable bolts are more widely used for their high mechanical properties, efficient reinforcement, flexibility, and convenient installation processes at low cost [3,4,5,6]. Typically, cable bolts consist of multiple high-strength steel wires wound together in a certain manner. High strength in cable wires, with the uniaxial tensile strength of approximately 1700 MPa, is achieved by the combination of using steels with higher carbon content and through manufacturing practices such as intensive cold-working and heat treatment. The cold-working manufacturing process creates the steel microstructures of the pearlite colonies and oriented wavy lamellae in the direction of cold drawing [7]. However, the cold-working also creates large dislocation density and vacancy concentrations in cable wires [8], which increases its susceptibility to SCC [9,10]. 

**Figure 1:**
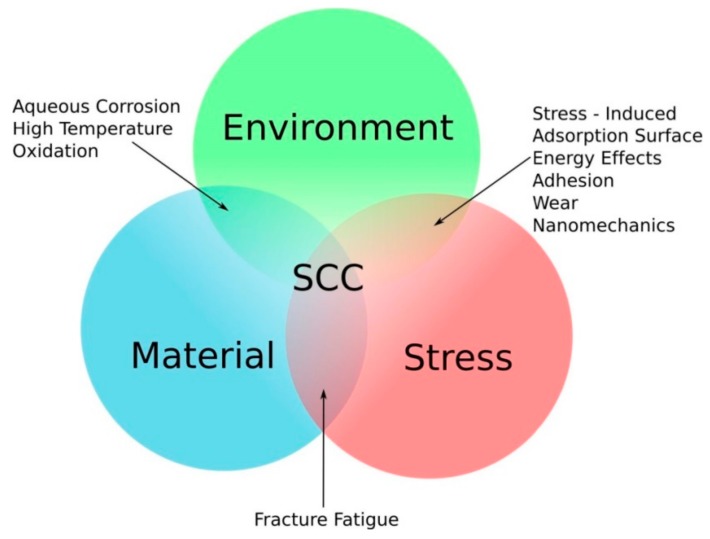
The relationship between stress, the environment, and susceptible material, modified from McCafferty [21].

In the past few decades, with the ubiquitous application of cable bolts at great mine depths, an increase in the frequency of cable bolts failures in reinforcement systems was observed. One of the causes of such failures was identified to be stress corrosion cracking (SCC). SCC is defined as the degradation process of material through the initiation and propagation of cracks under constant loads while exposed to corrosive environments [11,12,13,14,15]. SCC is a synergistic process that requires occurrence of three key elements: stress, an appropriately corrosive medium, and susceptible materials [16,17]. This synergy is described in the schematic shown in Figure 1. The conditions required to induce SCC vary depending on each of the key element. SCC is more threatening than traditional corrosion. A tiny crack can induce the failure of whole steel structures which have been simply overlooked in the past [18,19,20].

In underground reinforcement systems, SCC could induce the catastrophic failure of cable bolts, potentially leading to falls of ground. Generally, cable bolts are installed in predrilled boreholes using grout or cement, anchoring the weak layers to stable rock strata through the load-transfer capacity between bolt-anchor and grout interface. SCC failures of cable bolt were observed to occur in different geological and hydrological conditions. The SCC related rock bolt and cable-bolt failures in underground reinforcement system were first observed in the 1990s in the United Kingdom where the collapse of a roadway roof occurred [22,23]. SCC has also been identified as an issue in underground mines in China [24,25], Canada [26,27], Australia [15], and the USA [28,29]. However, only little research has been conducted to determine the long-term performance of cable bolts in underground conditions, particularly with regards to SCC. Spearing [28] conducted a series of laboratory tests to determine the effects of corrosion on the serviceability of cable bolts installed in underground coal mines in the USA. Different types of cable bolts including standard cable bolts, epoxy-coated cables, and galvanized cables were tested in a range of corrosive conditions with different pH and temperature. However, no SCC failure of the specimens was reported.

Maintaining integrity of the installed cable bolts during their service time is crucial for the sustainability and long-term stability of underground constructions. To fully understand the SCC and eventually avoid the occurrence of cable-bolt SCC in an underground reinforcement system, a dedicated testing system for investigating SCC in cable-bolt specimens was designed and constructed including long-term cable-bolt coupons and accelerated full-scale cable-bolt tension-loading apparatus. Both “long-term” tests using simulated underground environments and “accelerated” tests using an acidified solution were conducted. After the tests, the corrosion patterns and fractographic features of the specimens were observed and analyzed.

## 2. Experimental Programs 

### 2.1. Testing Specimens

**Figure 2:**
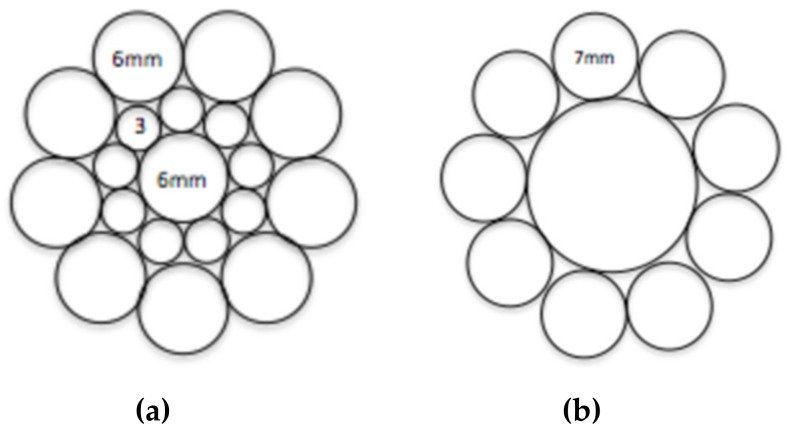
(**a**) Plain Superstrand (19 wires), and (**b**) TG bolts (9 wires/1central grout tube).

**Figure 3:**
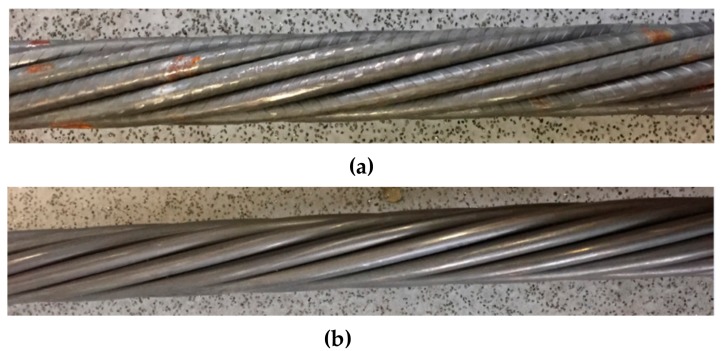
(**a**) Superstrand with indented wires, and (**b**) Superstrand with plain wires.

A wide range of cable bolts have been developed including plain cable bolts, bulbed cable bolts, and bird-caged cable bolts with both plain and indented wires. Different types of cable bolts are designed for application in different geological and geotechnical conditions. For example, the cable bolt with indented wires was designed to increase the bond strength between the cable and grout interface. The number of cable wires, wire arrangement, and geomoetry are different in different types of cable bolts. Superstrand and TG bolts are representive of the common characteristics of different types of cable bolts. Superstrand is solid and the TG is hollow. The nubmer of wires and wire arrangement of the cable bolts are shown in Figure 2. The pothograph of the Superstrand with plain and indented surface finish is shown in Figure 3. The mechanical properties of the Superstrand and TG bolts are provided in Table 1. 

**Figure 4:**
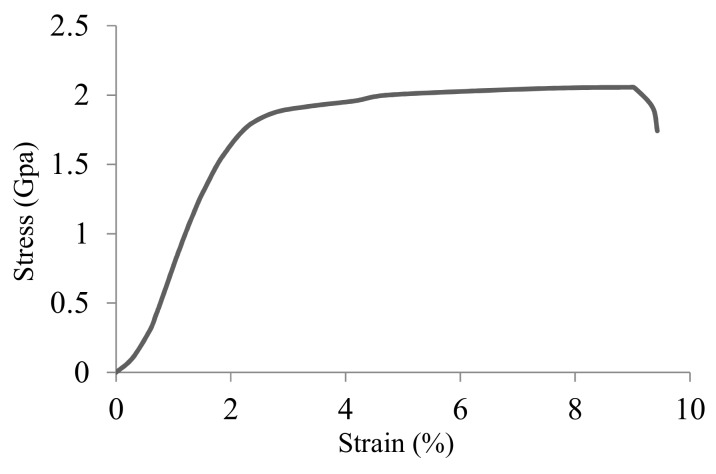
Stress–strain curve of the specimen obtained from a tensile test.

**Figure 5:**
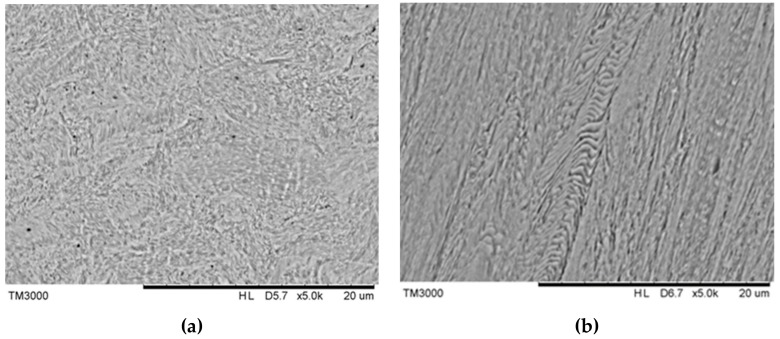
(**a**) Microstructure on cross-sectional direction, and (**b**) longitudinal direction.

Cable bolts consist of multiple individual wires that are wound together in a certain manner. To further characterize the material and mechanical properties of cable bolts, the chemical compositions and mechanical properties of the central straight wire of Superstrand with the diameter of 6 millimeters was analyzed (Table 2). The stress–strain curve of the specimen obtained from a tensile test and its microstructure are shown in Figure 4 and Figure 5. The microstructure on cross-sectional direction displayed the fine wavy pearlite lamellae (Figure 5a). In the longitudinal direction, the oriented wavy lamellae microstructure was clear (Figure 5b). The oriented wavy lamellae microstructure produced during cold-working manufacturing process provides the steel high mechanical properties over 1700 MPa of the ultimate tensile strength (UTS) [30]. However, as observed in Figure 5b, the cold-drawing manufacturing process also creates large dislocation density and large interlamellar spacing in ferrite lamellae, which increases its susceptibility to SCC.

ASTM G39 [31] recommends the use of flat, polished specimens for investigating the SCC failures in steels. However, the application of the polished specimens changes the surface conditions of the specimens. In this study, to preserve the integrity of the specimen surface profile, the central straight wires of Superstrand cable bolt were used for long-term cable-bolt coupon tests to characterize the effects of mineralogical materials on SCC. In terms of accelerated full-scale cable-bolt SCC tests, Superstrand and TG bolts made from indented and plain wires were used to study the SCC resistance of different types of cable bolts. 

### 2.2. Long-Term Cable-Bolt Coupon Tests

**Figure 6:**
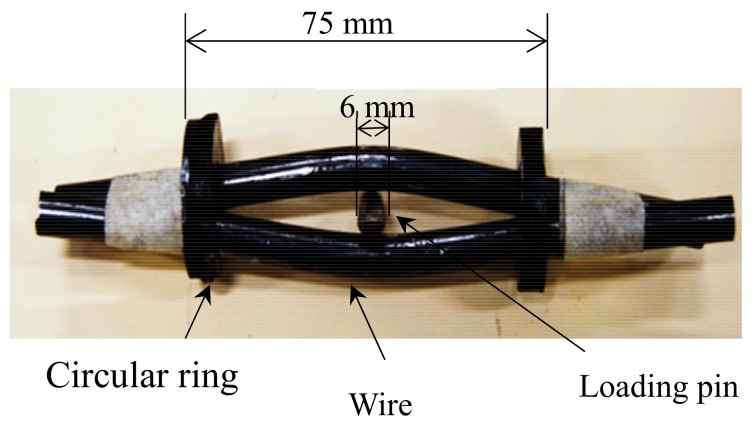
Photograph of the long-term cable-bolt coupon.

Cable-bolt SCC often occurred in the strata with clay bands and groundwater which suggested mineralogical-influenced corrosion was one potential factor causing the SCC failure [32]. Therefore, to determine the role of mineralogical materials in SCC failure, a novel three-point loaded cable-bolt coupon was designed, as shown in Figure 6. In the cable-bolt coupon arrangement, two wires were put in parallel and constrained at both ends, while the load was applied through the insertion of a loading pin. The loading pin was made from the same materials of the cable wire specimen. The tensile stress generated in outer fibers of cable wires simulated the real service stress conditions. The generated tensile stress in the specimen varied from zero at the constrained ends to maximum at the center loading point. Based on the analytical calculation, the maximum tensile stress in the cable-bolt coupon specimens was determined to be around 1600 MPa. In the preparation of the cable-bolt coupons, caution must be taken as slight inaccuracies in alignment of the wires and the pin would lead to an asymmetry in the amount of deflection experienced by each side of the specimen.

**Figure 7:**
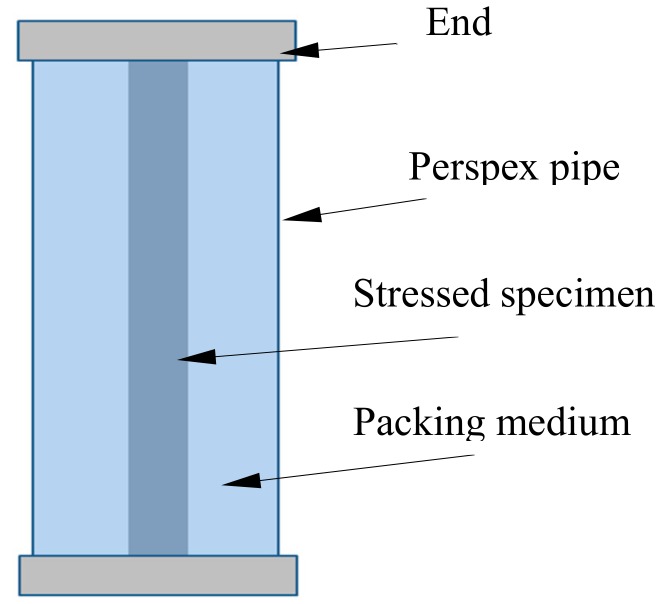
Long-term cable-bolt coupon test arrangements.

After the preparation of the long-term cable-bolt coupons, all the specimens were cleaned with acetone, labelled, and weighed. The loaded specimens were then immersed into the “corrosion cell” with different packing medium (Figure 7). The packing media were the groundwater, coal, and clay bands collected from the roadway strata of an underground mines where SCC of bolts occurred. Coal and clay samples were crushed to reduce the particle size until the average particle diameter was less than one centimeter. Three testing conditions were designed including the groundwater, clay bands saturated with groundwater, and coal–clay mixtures saturated with groundwater (Table 3). The specimens exposed to groundwater conditions were conducted as reference tests. The long-term cable-bolt coupon tests were conducted for 300 days which was considered to be an effective testing period for the occurrence of the measurable corrosion. The tests were conducted in the controlled mine environment laboratory at UNSW Sydney, Australia. Based on the environment characterization in two underground mines where SCC of cable bolts occurred, the temperature in the controlled mine environment laboratory was maintained at around 23 °C. To evaluate the corrosion process of cable-bolt specimens, one of the specimens was removed from each testing condition after exposure of 100, 200, and 300 days. The specimens were then cleaned using 10% acetic acid to remove the corrosion products [33], and weight loss due to corrosion was measured for each cable-bolt specimen. Corrosion patterns of the specimens in different test conditions were examined and compared.

### 2.3. Accelerated Full-Scale Cable-Bolt Tests

**Figure 8:**
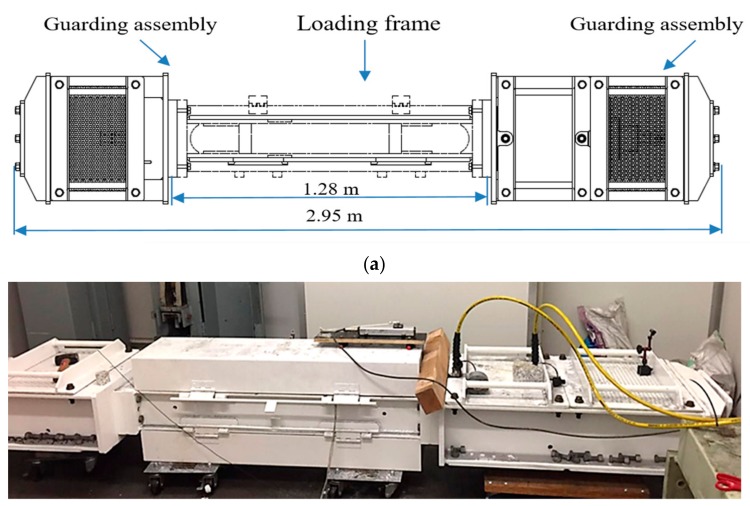
(**a**) Desigh of the tension-loading apparatus, and (**b**) a photograph.

To conduct SCC experiments on full-scale cable bolts, a tensile-loading apparatus was designed and constructed. The tensile-loading apparatus was composed of the loading frame, gripping system, and two guarding assemblies (Figure 8). The loading frame was composed of two parallel steel supports which were held together at the top and bottom by 8 cm thick steel endplates. These endplates were drilled to facilitate insertion and removal of cable bolts from the loading frame. The gripping system was used to restrain the cable bolt at both ends before introducing the tensile load. The guarding assemblies were mounted on each end of the loading frame for safety purposes. The tension-loading capability of the tensile-loading apparatus was more than 100 t.

**Figure 9:**
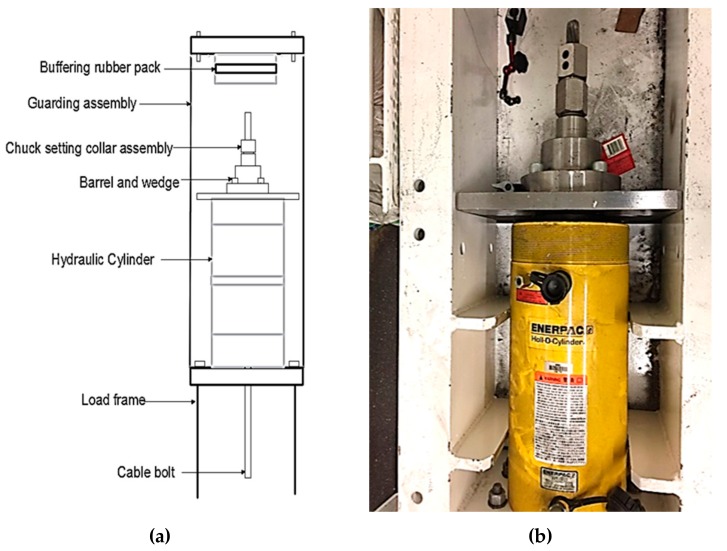
(**a**) Schematic view of the arrangement of the tension-loading system, and (**b**) a photograph.

In the tensile-loading system, a hydraulic cylinder and a mechanical nut retainment system were used to apply axial load to the cable-bolt specimens. The arrangement of the load frame, hydraulic cylinder, gripping system, and guarding assembly is displayed in Figure 9. An acidified solution was used as the testing solution, which was proved to be a reliable methodology to duplicate the service cable-bolt SCC failures in laboratory [34,35,36]. The chemical compositions of the solution were synthesized based on the groundwater chemistry of 12 underground mines where SCC of cable bolts occurred, as well as the characterization of localized conditions at the surface of cable bolt. The chemical compositions of the solution are shown in Table 4. Using this acidified solution not only accelerated corrosion process of high-strength steel but also provided a controlled and repeatable testing medium for SCC experiments. To maintain the condition of testing solution, a continuous cycling system was built in which the testing solution was collected and slowly cycled. A chemical cell made of flexible pipe was attached to the specimen inside the loading frame to facilitate the exposure and reticulation of the testing solution. Silicone sealant was applied to seal both ends of the chemical cell. During the test, the chemical solution was stored in a 5 L plastic container. Two reticulation pipes were used to connect the plastic container and the flexible pipe. Electronic pumps were coupled to the reticulation pipes to facilitate the flow of testing. The experiments were carried out in the controlled mine environment laboratory in which the temperature was maintained at around 23 °C.

The SCC experiments on Superstrands and TG bolts, made from plain and indented wires, were conducted under the periodically increasing strain-rate test conditions. In the periodically increasing strain-rate test, the axial deformation on the specimen was gradually increased at regular intervals for the duration of the test. Based on cable bolts’ mechanical properties, the axial strain rate applied on the specimens increased at 1.5% every day, while exposed to the acidified solution of two liters in the continuous cycling system. For each type of cable bolts, two cable-bolt specimens were tested to reduce the effect of scatter in the test results (Table 5). The acidified solution was refreshed with a new one every 24 h to maintain the aggressiveness of the acidified solution over the test duration. During the tests, the amount of the tensile load and displacement of cable-bolt specimen were recorded with the application of load cell and displacement sensors. The time to failure of each wire was recorded until the whole cable-bolt failure occurred.

## 3. Results and Analysis

### 3.1. Long-Term Cable-Bolt Coupon Tests

**Figure 10:**
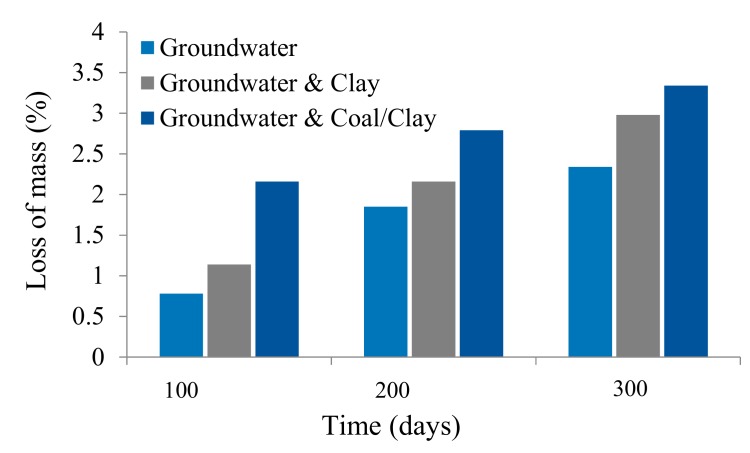
Weight loss of the specimens due to corrosion.

After exposure of 100, 200, and 300 days, one of the specimens was removed from each testing condition. The chemistry of the groundwater from each corrosion cell was analyzed after 300 days. The major cations and anion concentrations are illustrated in Table 6. Compared to the total dissolved solids (TDS) in the original groundwater, higher concentrations of TDS were observed in water samples from the corrosion cells packed with coal and clay. The pH of the groundwater sample was observed to be slightly alkaline. The weight loss due to corrosion was measured for each cable-bolt specimen (Figure 10). Generally, the specimens tested in the conditions of coal–clay mixtures had the largest weight loss, followed by the conditions of clay bands and the groundwater alone. The corrosion rate of the specimens decreased over time. This observation may account for the formation of a protective layer of iron oxides on the wire surface which prevented the diffusion aggressive medium to non-corroded sections. The weight loss of the cable bolts specimens in conditions of coal–clay mixtures was approximate double of that tested in groundwater alone, with the maximum weight loss at 3.34%. This demonstrated the significant effects of the mineralogical materials on accelerating the corrosion of cable bolts.

**Figure 11:**
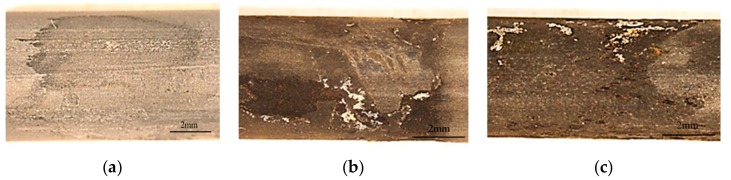
Corrosion patterns of cable-bolt specimens after 300 days, (**a**) groundwater, (**b**) clay with groundwater, and (**c**) mixture of coal and clay with groundwater.

Different corrosion patterns were observed in different testing conditions (Figure 11). Slight surface corrosion occurred on the cable-bolt coupons tested in the conditions containing groundwater alone. In contrast, severe localized corrosion was observed on the specimens tested in conditions of coal–clay mixtures. It should be noted that the localized corrosion was only observed at the middle section of the specimens where high stress was generated. This observation demonstrated the direct involvement of stress in accelerating the corrosion of cable bolts. The corrosion pits could accelerate the initiation and propagation of the micro cracks through generating local stress concentration at the bottom of the pits. Since the generation of SCC is a time-consuming process which requires significant exposure time [20], the occurrence of SCC failure may need longer testing duration. Using suitable synthetic solutions for accelerating and simulating the SCC failure in laboratory were recommended. These would assist in gaining further insights into the cable-bolt SCC through conducting relevant laboratory testing programs within sensible testing periods.

### 3.2. Accelerated Full-Scale Cable-Bolt Tests

**Figure 12:**
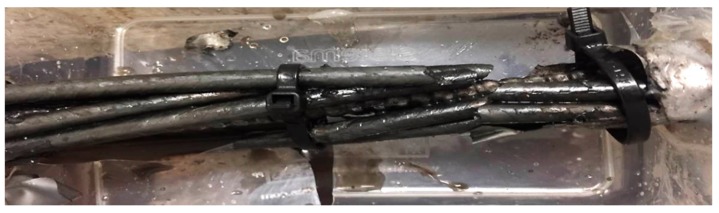
Failed TG bolts with indented wires.

**Figure 13:**
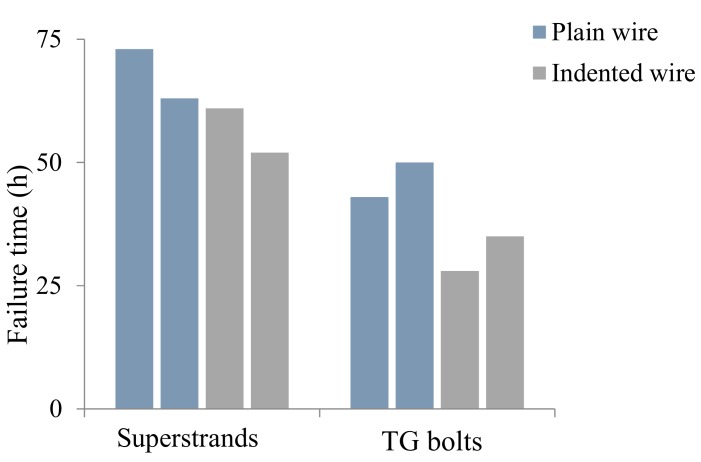
Time to failure of the first wire of full-scale cable-bolt specimens.

**Figure 14:**
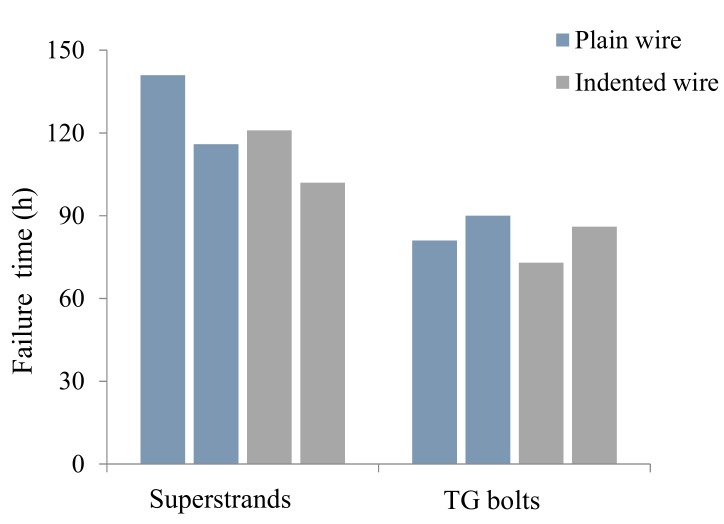
The failure time of the last wire of full-scale cable-bolt specimens.

All the full-scale cable-bolt specimens failed under the periodically increasing strain-rate test conditions while exposed to the acidified solution. An example of one failed TG bolt with indented cable wires is provided in Figure 12. The failure times of the first and last wire of the full-scale cable-bolt specimens were displayed in Figure 13 and Figure 14. Even though the time to failure of the wires in Superstrands and TG varied, the test results remained consistent with little scatter. For TG bolts with indented and plain wires, the failure times of the first wire were 31.5 and 46.5 h, respectively. Compared with TG bolts, the Superstrand showed higher resistance to SCC and the failure times of the first wire with indented and plain wires were 57 and 68 h. At the failure time of the last wire, the strains on the TG bolts with plain and indented were 7.5%. In terms of Superstrand, the strains on the cable bolt with indented wires were 7.5% at the failure time of last wire, while the strain was 9% for the cable bolts with plain wires. The higher strains observed on the Plainstrands compared to TG bolts at the failure time indicated higher resistance of Plainstrands to SCC than TG bolts. 

**Figure 15:**
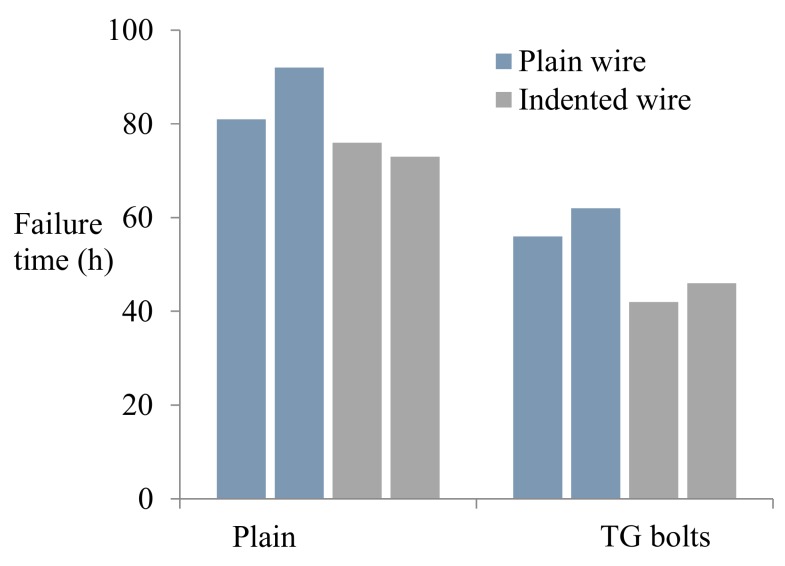
Average times to failure of the wires in full-scale cable-bolt specimens.

The average times to failure of all the individual wires are displayed in Figure 15. Similar to the failure times of the first wire and the last wire, the average times to failure of the same type of cable bolts with plain wires was approximately 25% longer than that with indented wires. For example, the average failure times of the cable wires in Superstrands and TG bolts with plain wires were 87 h and 59 h, while the average failure times were 75 h and 44 h for the intended cable bolts. Additionally, it was identified that Superstrand showed higher resistance to SCC than TG bolts of the same wire type under the same testing conditions. 

The designed tension-loading system together with the application of the periodically increasing strain-rate methods provided a reliable and efficient platform for the SCC examination of the full-scale cable bolts. The test results indicated the number of wires and wire surface conditions in different types of cable bolt have direct effects on SCC susceptibility. Generally, the cable bolts with a greater number of cable wires provided higher resistance to SCC. For the same type of cable bolts, the cable bolts with indented wires increased its SCC susceptibility. Based on the test results, cable bolts with a greater number of plain wires are recommended to be used in the areas with high risk of SCC. 

**Figure 16:**
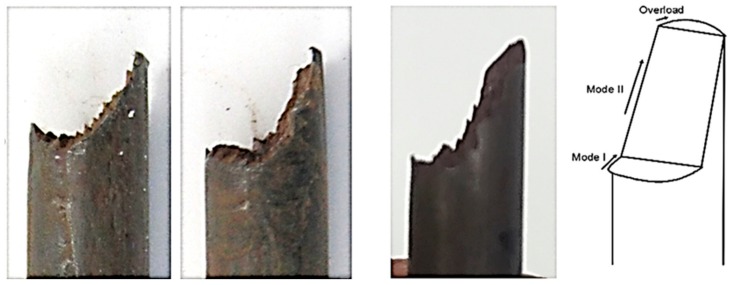
Step-shape fracture surface, Mode I tensile opening and Mode II shear opening.

The fracture characteristics of the failed cable bolts were examined to confirm the SCC occurrence. A typical step-shape fracture surface was observed on the cable wires from Superstrand with plain wires (Figure 16). It was identified that the fracture surface profile of the failed cable wires consisted of three crack growth modes (Mode I, Mode II, and Overload). The crack originally propagated into the wires in the direction perpendicular to the applied load through Mode I. After a certain length of crack propagation usually around one millimeter, the orientation of the crack propagation deflected and grew into the cable wire through Mode II. The step-shape fracture surface was widely recognized as a typical characteristic of SCC [37,38,39].

**Figure 17:**
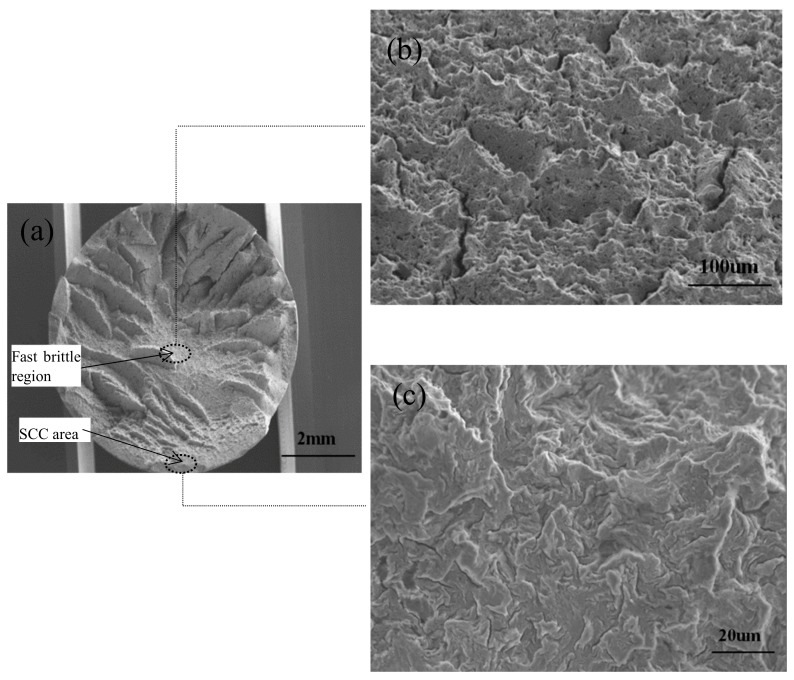
SEM micrograph of the failed cable wire, (**a**) overview, (**b**) ductile shear lip characteristics, and (**c**) TTS characteristics.

The surface fractographic features of the cable wires were examined using the scanning electronic microscope (SEM). The fractographic characteristics observed on the Superstrand and TG bolts with plain wires were similar and consistent. SEM images of the fracture surfaces from Superstrand with plain wires are presented in Figure 17. Three distinct regions were observed on the fracture surface. This observation was consistent with the crack propagation through tensile openings, shearing opening, and overload failure. The first region was found at the edge of the fracture surface characterized by a relatively flat area. The fractographic features in this region were typically characterized by the tear ridges appearance with certain degree of plasticity and closed spaced nucleation (Figure 17b). This observation was consistent with typical tearing topography surface (TTS) which is a significant characteristic of SCC [40,41,42]. 

**Figure 18:**
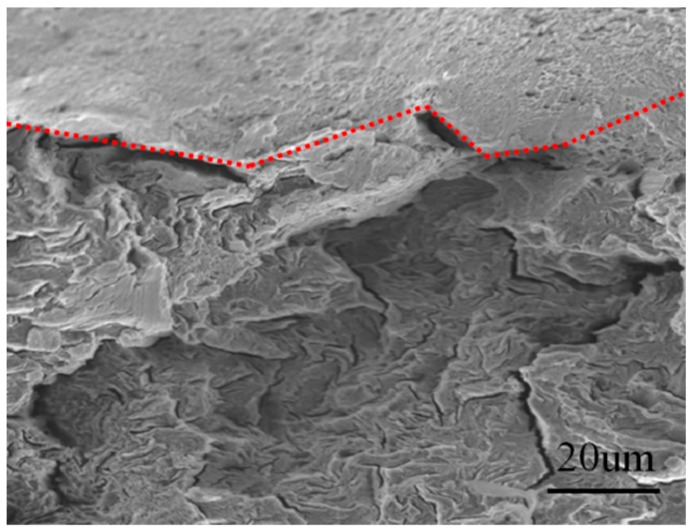
Transition between Mode I and Mode II.

The secondary region was following the SCC with some radial marks oriented towards this initiation area. Micro-voids and small ductile shear lip were observed in this region (Figure 17a). The transition in topography from SCC region to radial mark region was quite clear, as highlighted by the red line (Figure 18). The nearly straight transition line and different fractographic features indicated the crack propagated through different micro-mechanisms in Region I and Region II. The SCC crack propagated as a front through the Mode I tensile opening. After a certain length of crack propagation usually around one millimeter, the orientation of the crack propagation deflected occurred and propagated through the mode of shear opening. The third region was the overload region characterized by the shear lip. At this region, the crack propagated and grew to the critical size where the remaining cross-section could not support the load and overload failure occurred, inducing the shear lip.

**Figure 19:**
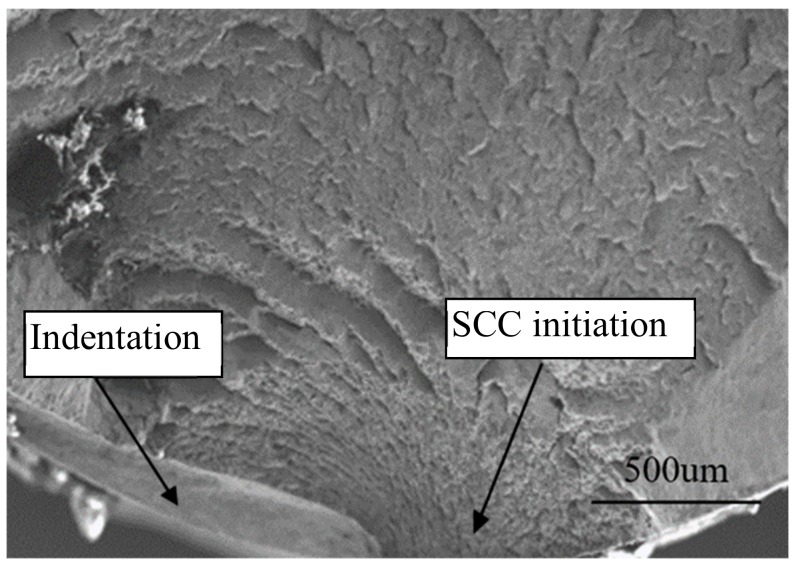
Crack initiation at the indented area.

It was noted that for cable bolt with indented wires, the crack initiated at the indented area (Figure 19). Even though the application cable bolts with indented cable wires improved the load-transfer ability through increasing the bond strength between grout and cable bolt, the indentation reduced the wire cross-sectional area and increased the stress concentration at the indented area. High stress at the indented area accelerated the initiation and propagation of SCC. In the areas with high risk of SCC occurrence, cable bolts with indented wires are not suggested to be used. This observation also explained the short failure time of the cable bolt with indented wires compared to cable bolts with plain wires under same testing conditions.

## 4. Conclusions

A two-step methodology was implemented, including long-term cable-bolt coupon tests using mineralogical materials collected from underground mine and an accelerated full-scale cable-bolt test using an acidified solution. The results showed that mineralogical materials and applied stress intensity accelerated the corrosion process of the cable bolts. Localized corrosion observed on the specimens could induce local stress concentration at the bottom of the pits and accelerate the initiation of the cracks. The cable-bolt coupons can be used for detailed investigations into cable bolts SCC through field experiments. The designed full-scale tensile-loading apparatus and periodically increasing strain-rate method proved a platform for examining SCC of full-scale cable bolt. The number of wires and wire surface conditions in different types of cable bolt directly affected its SCC susceptibility. The cable bolts with a greater number of wires provided higher resistance to SCC. In terms of wire surface conditions, the indented cable bolts were more suspectable to SCC compared to cable bolts with plain wires. The cracks usually initiated at the indented area. The indentation reduced the wire cross-sectional area and increased the stress concentration at the indented area, which accelerated the initiation and propagation of SCC. Even though the application indentation improved the load-transfer ability of cable bolts, cable bolts with indented wires are not recommended to be used in the areas with high risk of SCC. These results can be used to design optimal support systems in different environmental and geotechnical conditions.

## Figures and Tables

**Table 1:** Mechanical properties of Superstrand and TG bolts.

Types	Diameter	Yield Load	Elongation (Failure)
TG bolts	28 mm	568 kN	5–7%
Superstrand	21.8 mm	525 kN	5–7%

**Table 2:** Chemical composition and mechanical properties of the cable wire.

Chemical composition (wt.%)	C	Si	Mn	Ni	S	Cr	P
	0.85	0.31	0.66	0.02	0.02	0.11	0.013
Mechanical properties	Yield Strength		UTS		Elongation		Area reduction
	1600 MPa		1820 MPa		4.8%		28%

UTS, ultimate tensile strength.

**Table 3:** The long-term cable-bolt coupon testing programs.

ID	Packing Medium	Number
1	Groundwater	3
2	Clay and Groundwater	3
3	Coal/Clay and Groundwater	3

**Table 4:** Composition of the synthesized solution.

Solute	Mass (g/L)	Molarity (mol/L)
Sulfide sodium	1.5	0.019
Chloride sodium	0.45	0.008
Sulphate calcium	0.9	0.007
Acetic acid	25	0.42

**Table 5:** Full-scale cable-bolt tests.

Type	Cable Wires	Number
TG bolts	Plain	2
	Indented	2
Superstrand	Plain	2
	Indented	2

**Table 6:** The chemistry of the original groundwater and water samples after 300 days.

Specimen ID	Parameters (mg/L)						pH
	Ca	Fe	Cl	SO_4_	TDS	Dissolved Oxygen	
Original	14.2	139	7.4	80.5	398	5.92	7.82
1	2.26	10.30	26.0	52.6	339.72	3.81	9.11
2	49.92	5.12	12.0	513	956.30	6.06	9.63
3	19.90	0.18	27.5	140.7	1254.48	7.13	8.93
